# Reply: Validation of MELD 3.0 in 2 centers from different continents

**DOI:** 10.1097/HC9.0000000000000958

**Published:** 2026-05-22

**Authors:** Nikki Duong, Daniel J. Huynh, Newsha Nikzad, W. Ray Kim, Neil Shah, Scott W. Biggins, Allison Kwong

**Affiliations:** 1 Division of Gastroenterology and Hepatology, Stanford University, Stanford, California, USA; 2 Division of General Internal Medicine, University of Washington, Seattle, Washington, USA; 3 Section of General Internal Medicine, University of Chicago, Chicago, Illinois, USA; 4 Division of Gastroenterology and Hepatology, Mayo Clinic, Phoenix, Arizona, USA; 5 Division of Gastroenterology and Hepatology, University of North Carolina, Chapel Hill, North Carolina, USA; 6 Division of Gastroenterology, Hepatology and Nutrition, University of Pittsburgh, Pittsburgh, Pennsylvania, USA; 7 Stanford Transplant Outcomes Research Center (STORC), Stanford, California, USA

Dear Editors,

In July 2023, MELD 3.0 replaced MELD-Na for adult liver transplant allocation in the United States to reduce longstanding sex-based disparities in waitlist mortality by awarding females 1.33 additional points.^1^ Concurrently, the Organ Procurement and Transplantation Network (OPTN) replaced the demographic field “Gender” with “Birth sex” and introduced a new variable, “Sex for the Purposes of Adult MELD Calculation.” This policy change represents a critical step toward improving equity while acknowledging the presence of transgender and gender-diverse (TGD) individuals within the transplant population.

TGD individuals experience a disproportionate burden of risk factors for chronic liver disease, including viral hepatitis, alcohol-associated liver disease, and metabolic dysfunction.^2^ Data describing access to liver transplantation, allocation dynamics, and post-listing outcomes among TGD candidates remain extremely limited. The implementation of MELD 3.0 and accompanying OPTN data fields provides an opportunity to evaluate how allocation policies function for this historically understudied population.

To contextualize the early impact of these changes, we examined adult liver transplant candidates registered with OPTN between July 2023 and June 2025 who reported discordant entries for birth sex and sex used for MELD 3.0 calculation, comparing their characteristics and outcomes with those of cisgender candidates. Among 28,479 new adult registrations during this period, 96 candidates (0.3%) reported such a discrepancy.

There were no significant differences between TGD and cisgender candidates with respect to age at listing, race, insurance status, or liver disease etiology (Table 1). Native lab MELD 3.0 at listing was similar (20 vs. 18, *p*=0.98), even after excluding patients with MELD exception points within 90 days of registration (22 vs. 21, *p*=0.91). There were also regional differences—the geographic distribution of transgender liver transplant candidates based on the state of the listing transplant center (Figure 1). In sex-specific subgroup analyses, MELD 3.0 at listing was significantly higher in trans women than in trans men (21 vs. 16, *p*=0.01) (Table 2).

**TABLE 1:** Baseline characteristics and outcomes stratified by transgender status

Variable	No (n=28,475)	Yes (n=96)	*p*
Age, years, median (IQR)	56 (44–64)	57 (48–65)	0.50
Male sex at birth (%)	16,635 (58)	51 (53)	0.34
Race, n (%)			0.054
White	18,763 (68)	54 (60)	
Hispanic	5638 (20)	21 (23)	
Black	1755 (6)	4 (4)	
Other	1646 (6)	11 (12)	
Insurance, n (%)			0.34
Private	14,053 (49)	46 (48)	
Medicare	6562 (23)	26 (27)	
Medicaid	5169 (18)	12 (13)	
Other	2695 (10)	12 (13)	
MELD 3.0, median (IQR)	20 (14–29)	18 (15–29)	0.98
Etiology			0.15
Alcohol	11,205 (39)	42 (44)	
MASH	5572 (20)	21 (22)	
Viral	1322 (5)	8 (8)	
Autoimmune	2270 (8)	4 (4)	
Other	8110 (29)	21 (22)	
Exception case	5077 (18)	17 (18)	1.00
Region			0.009
1	1238 (4)	9 (9)	
2	2805 (10)	6 (6)	
3	4162 (15)	9 (9)	
4	3575 (13)	19 (20)	
5	4732 (17)	16 (17)	
6	883 (3)	2 (2)	
7	2192 (8)	14 (15)	
8	1692 (6)	3 (3)	
9	2004 (7)	8 (8)	
10	2236 (8)	4 (4)	
11	2960 (10)	6 (6)	
Outcomes			
LT (%)	18,013 (63)	57 (59)	0.50
Removal for death or too sick (%)	2228 (8)	6 (6)	0.70
Still waiting (%)	6395 (23)	30 (31)	0.053
Removal for other reasons (%)	1843 (7)	3 (3)	0.26
Days to LT, median (IQR)	19 (5–83)	11 (4–50)	0.03

Abbreviations: IQR, interquartile range; LT, liver transplantation; MASH, metabolic dysfunction–associated steatohepatitis; MELD 3.0: Model for End-stage Liver Disease 3.0.

**FIGURE 1:**
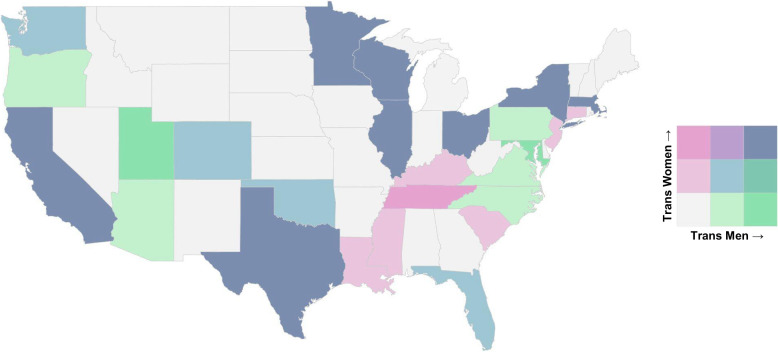
Geographic distribution of transgender patients listed for liver transplantation map of states categorized into tertiles by counts of trans men (*x*-axis) and trans women (*y*-axis). Colors represent combined tertile classifications, with darker shades indicating higher recipient counts. Grey denotes states with no recorded listing.

**TABLE 2:** Baseline characteristics and outcomes of transgender patients (FTM vs. MTF)

Variable	FTM (N=45)	MTF (N=51)	*p*
Age, years, median (IQR)	57 (48–66)	57 (48–65)	0.95
Race, n (%)			0.08
White	25 (58)	29 (62)	
Hispanic	8 (19)	13 (28)	
Black	1 (2)	3 (6)	
Other	9 (21)	2 (4)	
Insurance, n (%)			0.41
Private	24 (53)	22 (43)	
Medicare	12 (27)	14 (28)	
Medicaid	6 (13)	6 (12)	
Other	3 (7)	9 (18)	
Etiology			0.26
Alcohol	21 (47)	21 (41)	
MASH	8 (18)	13 (26)	
Viral	4 (9)	4 (8)	
Autoimmune	0 (0)	4 (8)	
Other	12 (26)	9 (18)	
MELD 3.0, median (IQR)	16 (11–27)	21 (17–31)	0.01
Exception case	11 (24)	6 (12)	0.18
Outcomes			
LT (%)	22 (49)	35 (69)	0.08
Removal for death or too sick (%)	5 (11)	1 (2)	0.15
Still waiting (%)	17 (38)	13 (26)	0.28
Removal for other reasons (%)	1 (2)	2 (4)	1.00
Days to LT, median (IQR)	23 (3–111)	8 (4–27)	0.36

Abbreviations: FTM, female-to-male; IQR, interquartile range; LT, liver transplantation; MASH, metabolic dysfunction–associated steatohepatitis; MELD 3.0, Model for End-stage Liver Disease 3.0; MTF, male-to-female.

Of the 63% of patients who had received a transplant by the end of follow-up, transgender candidates had shorter waiting times compared with cisgender candidates (11 vs. 20 days, *p*=0.02), despite similar allocation MELD 3.0 at transplant (27 vs. 27, *p*=0.64). However, when disaggregated, trans men (FTM) experienced longer waiting times for transplant and were more likely to be still waiting for transplant compared with trans women (MTF) and cisgender candidates. This supports the advantage provided to females with MELD 3.0, but suggests FTMs may be at a disadvantage in terms of transplant probability and mortality (Supplemental Figure S1, http://links.lww.com/HC9/C336).

Overall, demographic characteristics, disease etiology, insurance status, and laboratory-based MELD 3.0 scores at listing were similar between TGD and cisgender candidates, even after excluding individuals with early MELD exception points. These findings suggest that, in early implementation, the “Sex for the Purposes of Adult MELD Calculation” field is being applied in a manner consistent with its intended use rather than as a mechanism to gain an allocation advantage.

Although allocation MELD 3.0 scores at transplant were similar between groups, transgender candidates experienced shorter overall waiting times than cisgender candidates. Disaggregated analyses revealed opposing patterns: transgender women had higher MELD scores at listing and shorter waiting times, whereas transgender men tended to have lower MELD scores, longer waiting times, and a lower likelihood of transplantation during follow-up. When analyzed collectively, these opposing patterns were less apparent, underscoring the importance of avoiding aggregation across TGD subgroups.

These findings align with prior work by Tejedor et al.,^3^ which demonstrated improved prediction of short-term waitlist mortality for women under MELD 3.0 without disadvantaging men. At the same time, our observations highlight a key limitation of binary sex-based adjustments: clinically meaningful differences among TGD candidates may be obscured, particularly for transgender men, who in our cohort appeared to experience relative disadvantage. While MELD 3.0 represents a meaningful advance toward equity, sex-based adjustments alone may be insufficient to ensure fair allocation across all gender-diverse populations.

Encouragingly, we found no evidence of systematic misuse of MELD sex designation by transplant centers. The small proportion of candidates utilizing these fields likely reflects broader structural barriers to transplant access for TGD individuals, including discrimination, insurance instability, provider bias, and regional differences in access to affirming care.^4^

Moving forward, standardized and inclusive collection of sexual orientation and gender identity data, enhanced cultural competency, trauma-informed care within transplant programs, and continued research into the interplay between gender affirming hormone therapy and transplant implications will be critical. Ongoing evaluation of MELD 3.0 is necessary to ensure that efforts to correct historical inequities do not inadvertently create new disparities for transgender and gender-diverse candidates.

## Supplementary Material
