# Supplementary figures and images for: Reply: Validation of MELD 3.0 in 2 centers from different continents
Source: Hepatol Commun. 2026 May 22;10(6):e0958. doi: 10.1097/HC9.0000000000000958 (PMC13201025; doi:10.1097/HC9.0000000000000958)

Supplemental Figure 1.

Waitlist Mortality Stratified by Transgender Status

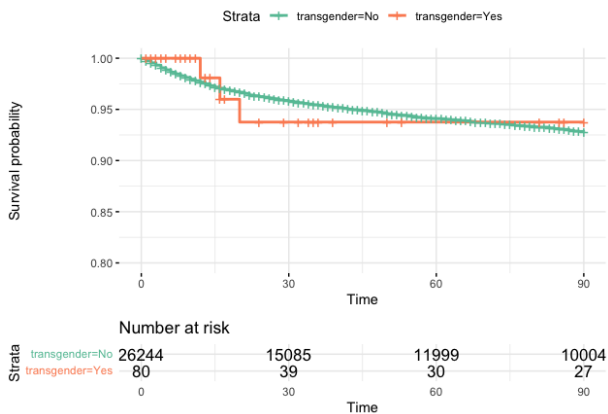

Supplement: Supplementary file 1 [file hc9-10-e0958-s001.pdf]
